# Checklist of vascular plant species on inselbergs in the Monumento Natural dos Pontões Capixabas, Espírito Santo State, Brazil

**DOI:** 10.3897/BDJ.12.e105688

**Published:** 2024-01-09

**Authors:** Fabiula Moreno Arantes, Luiza F.A. de Paula, Rafaela Campostrini Forzza

**Affiliations:** 1 Jardim Botânico do Rio de Janeiro, Rio de Janeiro, Brazil Jardim Botânico do Rio de Janeiro Rio de Janeiro Brazil; 2 Universidade Federal de Minas Gerais, Belo Horizonte, Brazil Universidade Federal de Minas Gerais Belo Horizonte Brazil; 3 Instituto Chico Mendes de Conservação da Biodiversidade, Prado, Brazil Instituto Chico Mendes de Conservação da Biodiversidade Prado Brazil

**Keywords:** taxonomy, floristics, rocky outcrops, granite, protected areas, rock mining

## Abstract

**Background:**

Inselbergs are granitic and/or gneissic rocky outcrops and, in Brazil, the dome-shaped ones in the Atlantic Forest Domain are called sugarloaves (*pães de açúcar*). They have an extremely specialised vegetation with high levels of endemism. Even though, they are poorly studied and highly degraded. In north-eastern Espírito Santo State, south-eastern Brazil, the *Monumento Natural dos Pontões Capixabas* (MONAPC) is a federal protected area created to guard some inselbergs mainly threatened by mining, which is one of the main economic activities in the State. In this work, we provide the first checklist of the vascular plant species in this protected area.

**New information:**

We recorded 108 species in 36 families and 75 genera that inhabit the vegetation islands on the inselbergs within the official limits of MONAPC. A new species of *Pleroma* (Melastomataceae) and a new species of *Cololobus* (Asteraceae) were discovered as new to science and they are being described in other articles.

## Introduction

Brazil is the country with the highest richness of vascular plant species in the world (Forzza et al. 2012). Around half of the species that occur in Brazil have been recorded in the Atlantic Forest, one of the most biodiverse (BFG [The Brazil Flora Group] 2021, Marques and Grelle 2021) and drastically decimated biomes in the world with only around 11% of the original forest remains (Ribeiro et al. 2009, Carlucci et al. 2021). The Atlantic Forest domain is a mosaic of different vegetation types and associated ecosystems (Scarano 2002) where inselbergs represent one of the frequent types of landscape. Inselbergs are enormous rock hills that rises abruptly from a plain (Twidale 1981, Twidale 1982, Porembski and Barthlott 2000, Porembski 2007, Varajão and de Alkmim 2015) and they are composed of granite and/or gneiss, occurring isolated or forming chains (Twidale 1981, Porembski 2007). They are present in other Brazilian Domains (Safford and Martinelli 2000, Barbosa-Silva et al. 2022) and comprise high species diversity in the eastern coast in a region called Sugarloaf Land (SLL) (de Paula et al. 2020).

Inselbergs, from the German words *insel* (= island) and *berg* (= mountain), are “terrestrial islands” characterised by their isolated and severe environmental conditions that are considered desert microclimates with high temperatures and insolation, a high rate of evapotranspiration and low humidity (Porembski 2007). When present, soil occurs as thin layers in depressions and on flat areas and is incapable of retaining rainwater that runs down the impermeable rock (Porembski and Barthlott 2000, Szarzynski 2000, Porembski and Watve 2005, Porembski 2007). Due to these extreme characteristics, the vegetation occurring on inselbergs sharply differs from that in surrounding areas and is an extremely specialised flora with a high number of endemic species (Porembski et al. 1998, Porembski 2007, de Paula et al. 2020).

In Brazil, there are few protected areas that include inselberg vegetation (Martinelli 2007, de Paula et al. 2020). The Monumento Natural dos Pontões Capixabas (MONAPC) is a protected area that includes the greatest concentration of inselbergs in south-eastern Brazil, it is within the SLL region in the State of Espírito Santo and still lacks biological inventories. A floristic list is fundamental when creating a management plan, a technical document that is primordial for an official protected area (Ministério do Meio Ambiente 2000). The management plan establishes guidelines and norms for conservation actions according to the objectives defined when creating the protected area. Thus, with the goals of contributing to sustainable management and decision-making, the objective of this work was to conduct a floristic inventory of the vascular plant species on the inselbergs within the legal limits of MONAPC.

## Project description

### Study area description

**Figure 1.**
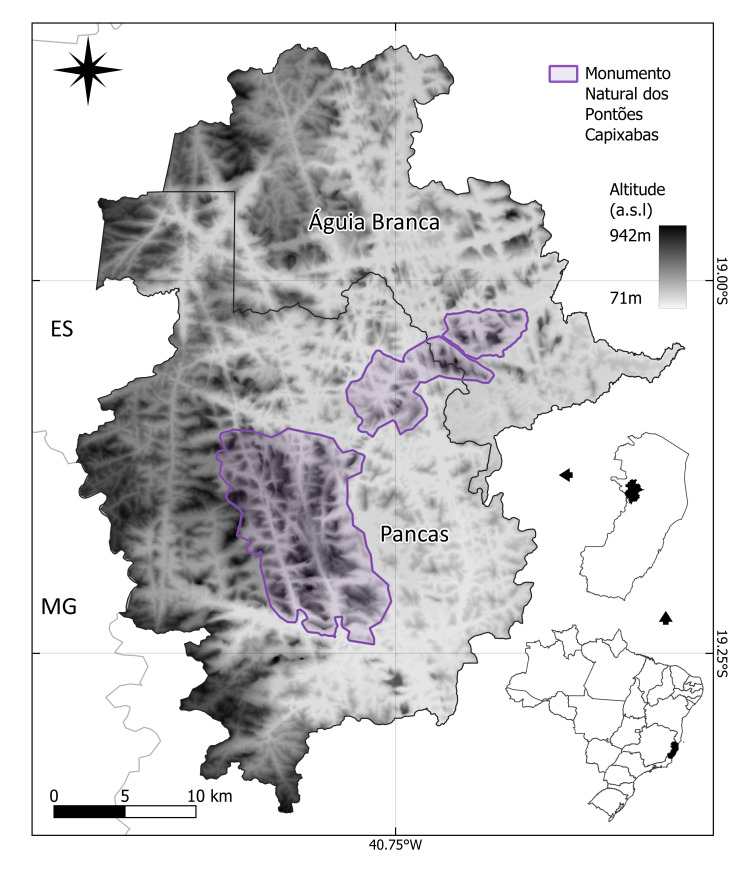
Location map depicting Pontões Capixabas Natural Monument, in the municipalities of Pancas and Águia Branca, Espírito Santo State, Brazil.

MONAPC is a federal protected area in Brazil. It is about 17,000 ha and is divided within two municipalities, 12,000 ha in Pancas and 5,000 ha in Águia Branca, in north-eastern Espírito Santo State (Fig. 1). In 2002, this protected area was created as a National Park to preserve the granite outcrops specially from mining, one of the most important economic activities to Espírito Santo State (Sardou Filho et al. 2013). The local population was technically not allowed to inhabit the region inside its legal limits (Ministério do Meio Ambiente 2000). Therefore, in 2008, the National Park was reclassified as a Natural Monument, a different category of protected area that allows people to inhabit inside its legal limits (Barbosa 2013, Bortoleto 2015, Spamer 2017).

MONAPC is divided into two microregions of the State, the Northeast Microregion and Central-West Microregion, which have various environmental problems. Both microregions bear the greatest number of municipalities in the State that are in a process of desertification, according to a national programme that was developed to assist extremely dry areas in Brazil (*Programa de Ação Nacional de Combate à Desertificação e Mitigação dos Efeitos da Seca* no Brasil) (Instituto Jones dos Santos Neves 2021). The Northeast Microregion comprises less than 8% of native forest, less than 1% of it being within protected areas and around 60% of pasture, which is the highest percentage in the State (Instituto Jones dos Santos Neves 2021). The Central-West Microregion has circa 12% of native forest, less than 3% of it being officially protected and the main soil use is for coffee crops (around 16%) (Instituto Jones dos Santos Neves 2021). For the two microregions together, around 11% of the agricultural areas have degraded soils, around 18% of the coffee crops have degraded soils and around 21% of the pastures are degraded (Barreto and Sartori 2012). The rural properties within the legal limits of the protected areas are small and mostly specialised in cultivating robusta coffee (*Coffeacanephora* Pierre ex A. Froehner) (Ferrão et al. 2019) or farming cattle (Instituto Jones dos Santos Neves 2021).

**Weather**

**Figure 2.**
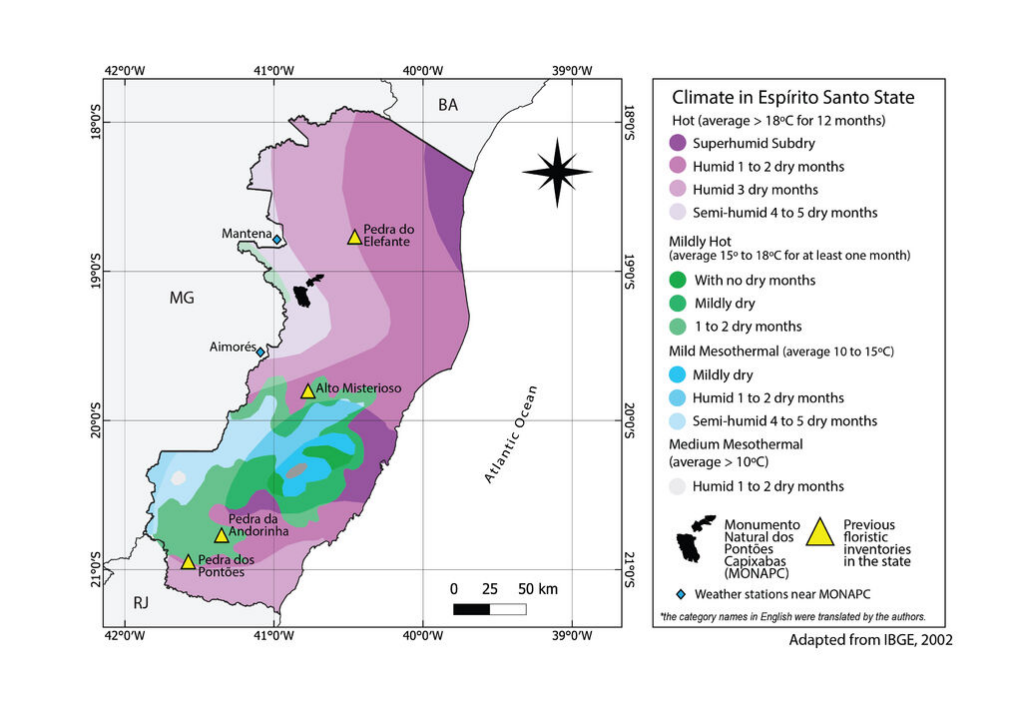
Climate map and location of previous studies about inselbergs and weather stations near the present study area, Espírito Santo State, south-eastern Brazil.

The Brazilian Institute of Geography and Statistics (Instituto Brasileiro de Geografia e Estatística 2002) classifies the climate of northern Espírito Santo as hot and dry. In a more general classification by Köppen (1936), MONAPC has a tropical climate with a dry winter and rainy summer (Aw) (Fig. 2).

**Figure 3.**
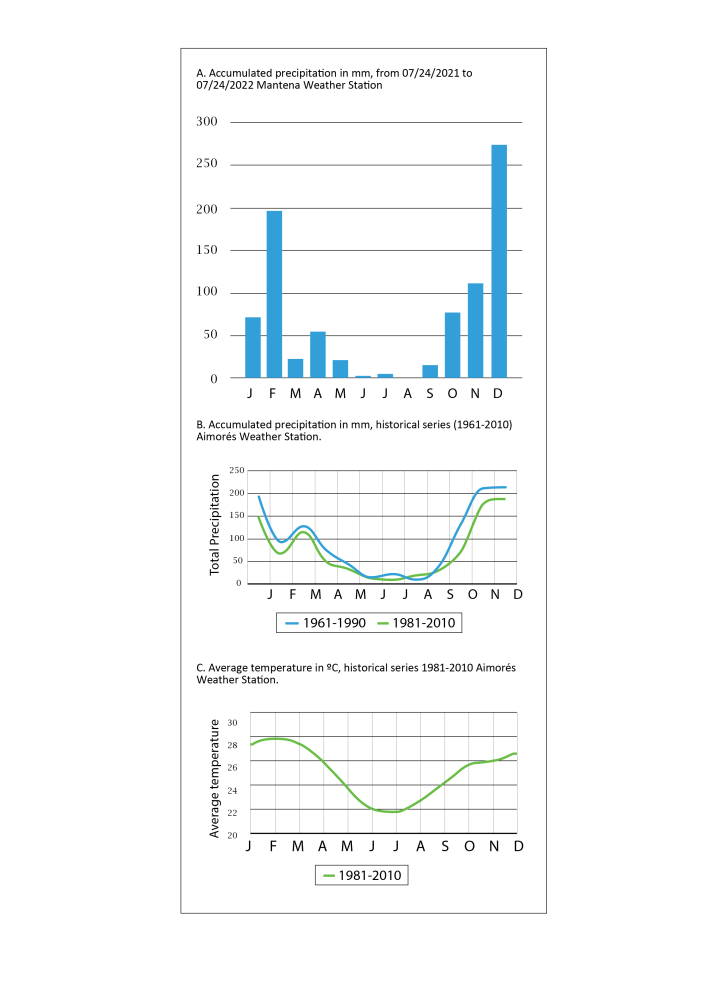
Climate data from two weather stations near the Monumento Natural dos Pontões Capixabas, Espírito Santo State, Brazil.

The weather stations nearest to MONAPC are Aimorés (A534 -19,532778, -41,090833; 287,74 a.s.l.), around 30 km to the southeast and Mantena (A540 -18,780620, -40,986505; 254,91 a.s.l.), around 40 km to the northeast (Fig. 2). Data from the Mantena station showed that the average temperature of the coldest months (June to August) is 21ºC and the sum of the precipitation for the period of 92 days is less than 10 mm. During the hottest months (January to March), the average temperature is 27ºC and the precipitation increases considerably to nearly 300 mm for the entire period. During this work, the rainiest months were from October 2021 to February 2022; in December, there was 277 mm of rain (Fig. 3, A). Graphs of the precipitation and average temperature from the Aimorés station (which were not available for the Mantena station) demonstrate a climate pattern that is very demarcated for the region, with dry winters and rainy summers (Fig. 3, B and C).

**Geology**

Inselbergs are very old outcrops formed underground and revealed by weathering on the surface (Varajão and de Alkmim 2015). They can be various types of rock and occur in different climates, but the inselbergs in MONAPC are made of granite and gneiss from the crystalline core of the *Araçuaí* orogen, formed during the separation of Gondwana at the end of the Neoproterozoic and beginning of the Paleozoic (Varajão and de Alkmim 2015). The three types of rock that form the inselbergs in MONAPC are *Ataléia* and *Carlos Chagas* granites (575 m.y.a.) and Charnockito *Aimorés* (520 to 490 m.y.a.) (Instituto Brasileiro de Geografia e Estatística 1987). The most frequent is *Carlos Chagas* leucogranite, which is formed by large crystals of feldspar in a matrix of plagioclase, quartz and garnets (Varajão and de Alkmim 2015).

Inselbergs are classified as lowlands when they are up to 1000 m above sea level (*sensu* de Paula et al. 2016) and highlands when they exceed this height (*sensu* Safford (1999)). Average elevation in Pancas is 110 m to 480 m and, in Águia Branca, around 278 m.

In MONAPC, 195 outcrops have been recorded (OpenStreetMap Foundation 2022) that are up to 1000 m a.s.l., so they are considered lowlands. The region predominantly has red-yellow latosols (halic or dystrophic) with a clayey texture associated with the crystalline rocks of the inselbergs (Instituto Brasileiro de Geografia e Estatística 1987).

## Sampling methods

### Study extent

MONAPC and its surroundings are in the Atlantic Forest domain and the main forest formation in the region is Semideciduous Seasonal Forest (Instituto Brasileiro de Geografia e Estatística 2012), which encircles the inselbergs (Saiter et al. 2021). Fragments of Riverine Forest (Instituto Brasileiro de Geografia e Estatística 2012) also occur along streams in the region. On the rock surface of the inselbergs, there is a mosaic of vegetation types organised into vegetation patches or islands surrounded by bare rock. Here, we use the term vegetation island to designate diverse microhabitats on the inselbergs that are totally exposed to the xeric conditions of these environments, from fissures and hollows in the rock to mats of monocotyledons directly fixed to bare rock (Porembski et al. 1997, Seine et al. 2000). The focus of our study was the vegetation islands directly exposed to the harsh enviroment of inselbergs (Seine et al. 2000). The vegetation islands mainly comprise plants forming mats, such as species of Velloziaceae, Bromeliaceae and Cyperaceae (Porembski and Barthlott 2000) that grow directly on bare rock or thin layers of substrate. Other vegetation types in MONAPC were also visited, such as scrub vegetation with shrubs and trees (Rizzini 1997, Oliveira-Filho 2013). These are usually found on less steep slopes of the inselbergs, which favour sedimentation that results in shallow soil with forest formations. Scrub vegetation is very common on the flat ridges of some inselbergs, in depressions on the rock surface and in transition areas between the forest matrix and exposed rock. Some occasional collections made inside forest vegetation were not included on the final list.

### Sampling description

Vegetation islands were sampled between September and November 2021, covering the dry and rainy seasons. On the first expedition in MONAPC, we drove on dirt roads amongst the 195 mapped inselbergs (OpenStreetMap Foundation 2022) to look for those with less steep slopes that could be safely accessed without using climbing equipment. During the preliminary analysis of digital records from the region, we found a lack of collections from within the official limits of the MONAPC. Therefore, we decided to visit as many mapped points as possible on the first expedition. We returned to some of the inselbergs whenever possible to make additional collections (depending mostly on the weather), but some points were only visited once.

The collected material was preserved in alcohol (70%) until it arrived in the herbarium where it was processed (Mori et al. 1989). All the collections from the authors were deposited in the RB Herbarium and duplicates were sent to the VIES and SPF Herbaria; some duplicates were also sent to specialists at the HUEFS, CEN, UPCB and HUFU Herbaria (acronyms according to Thiers (2023)). Leaf fragments were separated and dehydrated in silica before preserving the collections in alcohol. These samples were deposited in tissue collection at RB Herbarium (Instituto de Pesquisas Jardim Botânico do Rio de Janeiro 2023).

The specimens were identified by comparing them with identified material at RB, consulting taxonomic articles, consulting keys in Flora e Funga do Brasil (2023) and sending photos and/or duplicates to specialists. Taxonomic names follow Flora e Funga do Brasil (2023) and also recent papers published by specialists. All the taxa present in the list have their unique herbarium voucher and we selected only vouchers determined by their respective taxa specialists.

Information about life form, substrate, vegetation type, domain and occurrence in federative units were taken from Flora e Funga do Brasil (2023) using the interface PlantMiner (Carvalho 2023). Flora e Funga do Brasil (2023) follows Angiosperm Phylogeny Group (2016) and The Pteridophyte Phylogeny Group (2016). The endemic species from the granite inselbergs in southeast Brazil were determined using distribution data mainly taken from Flora e Funga do Brasil (2023) and/or by consulting papers about species description and their distribution (Table 6).

All analyses with the occurrence databases were conducted in the R programming language (R Core Team 2022) with the development software R Studio (R Studio Team 2022) and the software Excel® (Microsoft® 2022). The following R packages were used to manipulate data: *plyr* (Wickham 2011), *dplyr* (Wickham et al. 2022), *magrittr* (Bache and Wickham 2022) and sqldf (Grothendieck 2017).

**Previous Floristic Inventories**

We compared the results of this work with other studies conducted on inselbergs in Espírito Santo State: Esgario et al. (2009),Couto et al. (2017), Pena and Alves-Araújo (2017) and Covre et al. (2021)(Table 1).

### Step description

**Vascular Plant Dataset**

**Figure 4.**
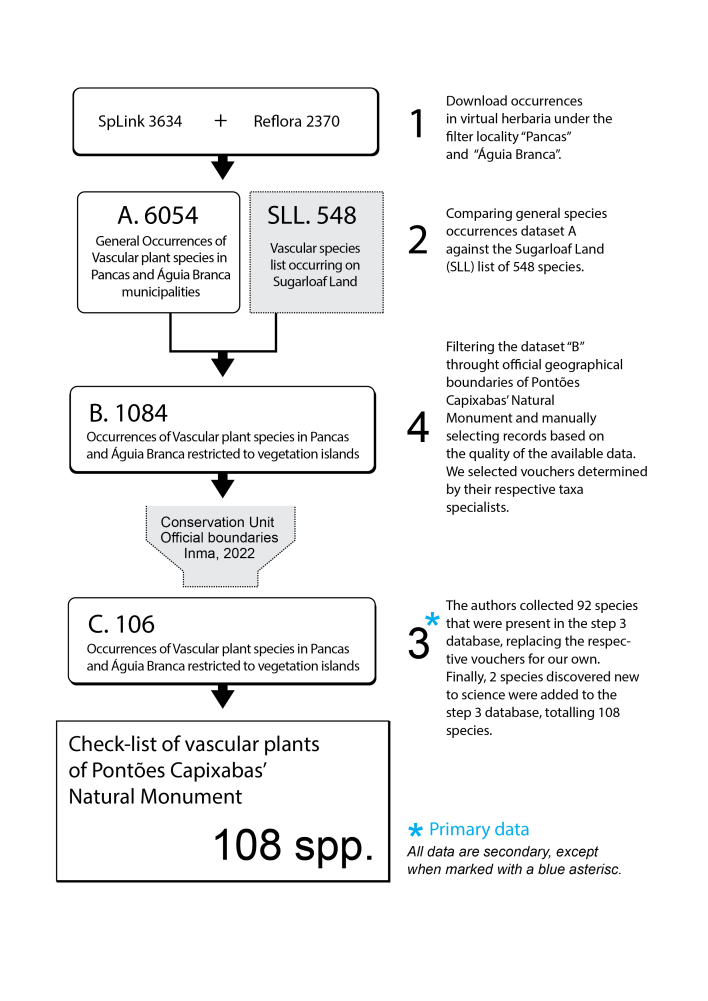
Construction and validation of vascular plant dataset for inselbergs in the Monumento Natural dos Pontões Capixabas, Espírito Santo State, Brazil.

The list of species was constructed in four steps (Fig. 4):

1. We compiled all the occurrence records of vascular plant species from the municipalities of Pancas and Águia Branca, which were in the Reflora Herbário Virtual (2022) and Herbário Virtual da Flora e de Fungos (Centro de Referência e Informação Ambiental 2022) online databases. At the end of this step, we had 6,054 records (including duplicates) of 1,180 species.

2. A comparison was made between the species in SLL (which has 548 vascular plant species on vegetation islands; de Paula et al. (2020)) and the database of the species compiled in step 1. Only records of corresponding species were kept, thus ensuring which species occur on vegetation islands. Of the 6,054 records, we kept 1,084 records of 184 species from vegetation islands (and occasionally scrub vegetation).

3. We found 106 records restricted to the official geographic limits of MONAPC (Instituto Chico Mendes de Conservação da Biodiversidade 2023) and determined by their respective taxa specialists.

4. The authors collected 92 species that were present in the step 3 database, replacing the respective vouchers for our own. Finally, two species discovered new to science were added to the step 3 database, totalling 108 species in the first “Checklist of the Vascular Plant Species in MONAPC”.

## Geographic coverage

### Description

The geographic coverage encompasses lowland inselbergs in the Monumento Natural dos Pontões Capixabas (MONAPC), a federal protected area in north-eastern Espírito Santo State, south-eastern Brazil. Ten inselbergs were visited (Table 2, Fig. 8).

### Coordinates

-19.245509, -40.766437 and -19.000903, -40.866024 Latitude; -19.102962, -40.868911 and -19.018078, -40.661117 Longitude.

## Taxonomic coverage

### Description

We provide the first “Checklist of Vascular Plant Species in MONAPC”, which has 108 species distributed in 36 families and 75 genera (Suppl. material 1, Fig. 10). Although we found a richness of 108 species in MONAPC, other authors found higher numbers in other inselbergs: Covre et al. (2021) found 121 species on Pedra das Andorinhas, Couto et al. (2017) found 211 species on Pedra dos Pontões, Pena and Alves-Araújo (2017) found 302 species in the APA Pedra do Elefante and Esgario et al. (2009) found 170 species in Alto Misterioso (Table 3). The low number of species found in MONAPC compared to the other floristic works conducted on inselbergs in Espírito Santo State could be due to differences in the methodology (as previously mentioned, we only sampled vegetation islands, while the other studies also sampled other vegetation types) and/or the floristic composition of each rock outcrop.

The angiosperm lineage is the richest, with 98 species distributed in 70 genera and 32 families. The richest families are Bromeliaceae (13 spp.), Asteraceae (11 spp.), Melastomataceae (9 spp.), Orchidaceae (7 spp.), Araceae and Apocynaceae (5 spp. Each) and Fabaceae (4 spp.). Together, these families represent 79% of all the species on this list. The richest genera are *Pleroma* (6 spp.) and *Anthurium* (4 spp.), followed by six genera with three species each: *Anemia*, *Cololobus*, *Dioscorea*, *Dyckia*, *Mandevilla* and *Selaginella*. Thirteen genera have two species each and 54 genera are represented in the local flora by only one species (50%) (Fig. 9).

There are 10 species of lycophytes and monilophytes. The lycophytes are only represented by Selaginellaceae (*Selaginella*, 3 spp.), while the monilophytes are represented by seven species and three families, Anemiaceae and Pteridaceae with three species each and Blechnaceae with one species. The richest genus is *Anemia* (3 spp., Anemiaceae), followed by *Cheilantes* (Pteridaceae) with two species and *Doryopteris* (Pteridaceae) and *Blechnum* (Blechnaceae) with one species each.

Most of the species found in this study have an exclusively herbaceous life form (48 spp.; 50%), followed by shrubs (16 spp.; 17%), vines and subshrubs (5 spp. each; 5%), trees (5 spp.; 4%) and one species of palm (*Syagrusruschiana* (Bondar) Glassman). Seventeen species have more than one life form (10%) (Fig. 9, Suppl. material 1).

The most species-rich families in this study are the same as those in other works conducted on inselbergs in Espírito Santo. The richest family was Bromeliaceae, as found by Pena and Alves-Araújo (2017) and Covre et al. (2021); however, this was the second richest family in Couto et al. (2017) (Fig. 7). Asteraceae and Melastomataceae ranked amongst the first four positions in all the works, including the present study (Fig. 7). According to Porembski et al. (1997) and Porembski and Watve (2005), Bromeliaceae, Orchidaceae and Melastomataceae are typical families of inselbergs in the southeast region of Brazil.

The richest families on the inselbergs in MONAPC are also the most diverse in the Atlantic Forest domain in Espírito Santo State. These families are Orchidaceae, Bromeliaceae, Fabaceae, Asteraceae, Myrtaceae, Rubiaceae, Melastomataceae, Apocynaceae, Cyperaceae and Poaceae (Dutra et al. 2015). These are also amongst the most representative families on inselbergs in Sugarloaf Land (de Paula et al. 2020) and alternate amongst the top positions, which reinforces the connection between the species assemblages in the different ecosystems and phytophysiognomies in the Atlantic Forest domain (Scarano 2002, Neves 2017). Species in the forest matrix colonise inselbergs and those in temporary refuges on inselbergs (e.g. during cycles of environmental changes) also return to the matrix (Burke 2002a, Burke 2002b, Burke 2003, Porembski 2007). Thus, as considered for ocean islands (e.g. Heaney (2007)), it is believed that populations on inselbergs can be sources or sinks, depending on the environmental niche of the species (Burke 2003). Long-term monitoring of selected species populations, dated phylogenies and biogeographic approaches are needed to better explain if “source-sink” effects exist and how they operate.

There were 33 exclusively rupicolous species and 32 exclusively terrestrial species. Twenty-one species were both rupicolous and terrestrial. Only one species was exclusively epiphytic (*Stigmatodonvellozicolus* (Leme & J.A.Siqueira) D.R.Couto & A.F.Costa), while seven species were epiphytic and rupicolous. Finally, combined substrates, rupicolous/hemi-epiphytic and aquatic/terrestrial, had only one species each. Interestingly, Bromeliaceae and Orchidaceae are generally the richest families in the Atlantic Forest and mostly represented by epiphytes in forest physiognomies, but on inselbergs, they are represented by rupicolous groups. It is speculated that vegetation richness in epiphytes in the Atlantic Forest region (Benzing 2000, Givnish et al. 2014) could influence the high richness of bromeliad species on inselbergs in south-eastern Brazil, or vice versa, especially for Tillandsioideae (de Paula et al. 2016). This can culminate in the evolution of species that are efficiently adapted to the severe environmental conditions in canopies and on rocky outcrops (Porembski et al. 1998). More studies are needed to understand to what extent epiphytic bromeliads (of a regional pool of species) share preferences for a similar habitat with rupicolous elements of inselbergs.

Amongst the species recorded in MONAPC, fifteen are on the Brazilian Red List (Ministério do Meio Ambiente 2022) under one of the threatened categories (3 CR, 6 EN, 6 VU) and 25 are on the Espírito Santo State Red List (Espírito Santo 2022) under one of the threatened categories (5 CR, 9 EN, 11 VU). The family with the greatest number of threatened species is Bromeliaceae (8 spp.), followed by Melastomataceae (5 spp.), Orchidaceae (4 spp.) and Asteraceae and Anemiaceae (2 spp. each). Each of the remaining 12 families have one threatened species (Table 4). Only five species are classified in the same categories on both lists: *Orthophytumzanonii* Leme (CR), *Kielmeyerarupestris* Duarte (CR), *Stigmaphylloncrenatum* C.E.Anderson (CR), *Meriantherapulchra* Kuhlm. (VU) and *Epidendrumrobustum* Cogn. (VU).

**Nomenclatural types**: According to the Reflora and SpeciesLink virtual herbaria, 29 species of plants were described from specimens collected in Pancas and Águia Branca over the last 80 years (Table 5). The oldest type collections are from 1942, *Ixoraemygdioi* (E.A. Bruno n. 191) and 1977, *Mandevillagrazielae* (G.J. Sheperd n. 5869), which is a typical species of inselbergs and were described 70 and 29 years after being collected for the first time, respectively. For 20 years, there were no collection records in the region and, in 2003, two new species of Melastomataceae were collected and described 11 and 12 years later. The highest number of types were collected in 2006 (11 collections), of which some were described a decade later. In 2007 and 2008, there were five species each and, in 2010, there were two species. After a hiatus of 5 years without type collections, 2016 and 2017 each had one collection. The time for a species to be described is highly variable and it depends on the specialists in the families. Eighteen species took 5 to 13 years to be described, while 14 species took less than 5 years.

**New records within the official limits of MONAPC**

*Orthophytumzanonii* was only known from two records from the “*Pedra do Vidal Krause*” inselberg, which is a popular tourist spot in the region. In this work, we recorded another occurrence of this species on an inselberg on private property. It is important that these records were made within a protected area because this ensures the plants are protected at least from mining, which is the greatest threat to inselbergs.

Thirty-eight species in the checklist are endemic to granite rocky outcrops in southeast Brazil (Table 6). Some genera are typical of inselbergs, such as *Cololobus* and *Wunderlichia*, from the Asteraceae family and *Merianthera*, from the Melastomataceae family (Safford and Martinelli 2000, Fig. 5).

Widely-distributed species were also collected on the inselbergs in MONAPC and are included on the list. These species were collected to help studies about species migration from the surroundings to the vegetation islands. Inselbergs are inadequate for agriculture and pastures, but around them, coffee plantations and pastures have replaced the forest matrix. The pastures and crops directly touch the rocks and, in some cases, there is a transitional vegetation comprising shrubs, vines, grasses and other herbs.

We collected *Melinisrepens* (Willd.) Zizka (Poaceae) on all the inselbergs visited. This is an African grass considered invasive (Instituto Brasileiro de Geografia e Estatística 2012) and has already been described as a serious threat to Brazilian inselbergs (de Paula et al. 2015, Porembski et al. 2016). The Flora e Funga do Brasil (2023), however, classifies this species as naturalised, according to Pyšek et al. (2004).

**Gap in collections within the official limits of MONAPC**

**Figure 6.**
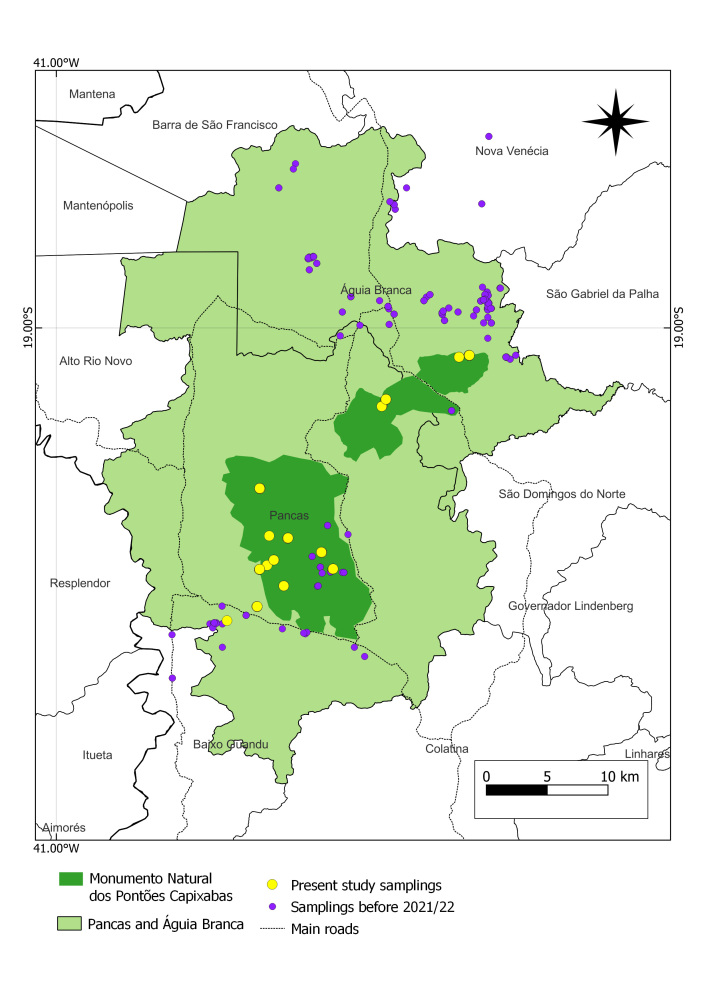
The sampling gap within the official limits of the Monumental Natural dos Pontões Capixabas (MONAPC), Espírito Santo State, Brazil.

**Figure 7.**
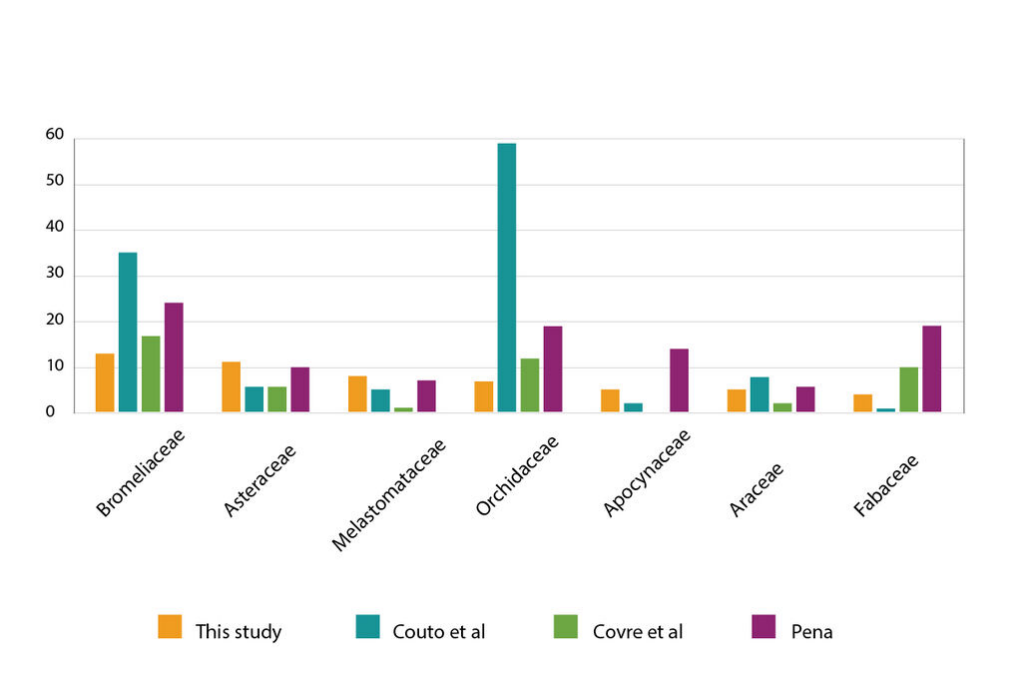
Comparison of family richness between the present and the previous studies on inselbergs from Espírito Santo State.

**Figure 8.**
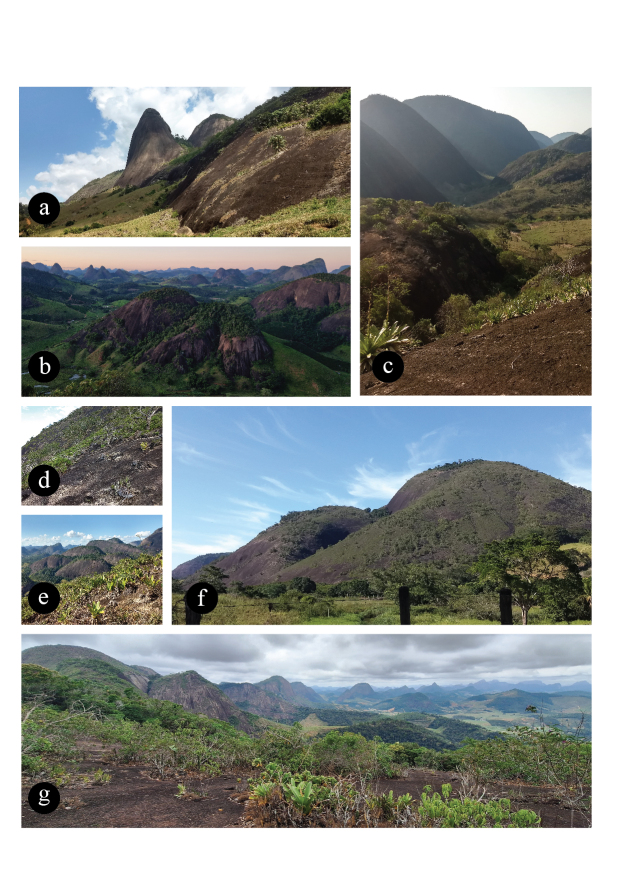
Some Inselbergs visited in MONAPC, Espírito Santo State. **a** Paredão das Ruschianas; **b** Mirante's view (northeast); **c** Paredão Noroeste's view (northwest); **d** Bromeliaceae garden at Mirante (northeast); **e** Velloziaceae garden at Mirante; f Pedra do Vidal Krause's view.

**Figure 9.**
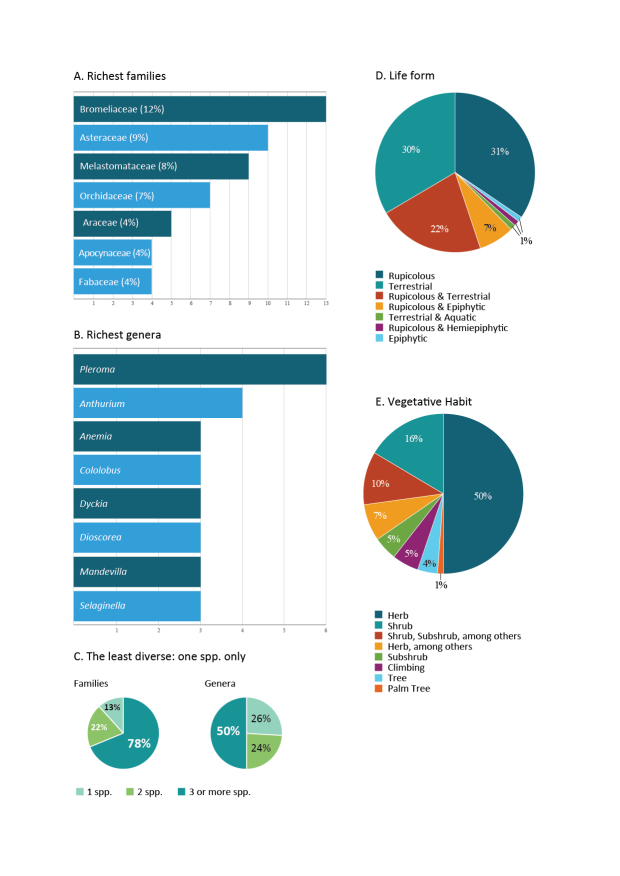
Richest families and genera, least diverse families and genera, life forms and vegetative habits in the Monumental Natural dos Pontões Capixabas, Espírito Santo State, Brazil, according to Flora e Funga do Brasil (2023).

**Figure 10.**
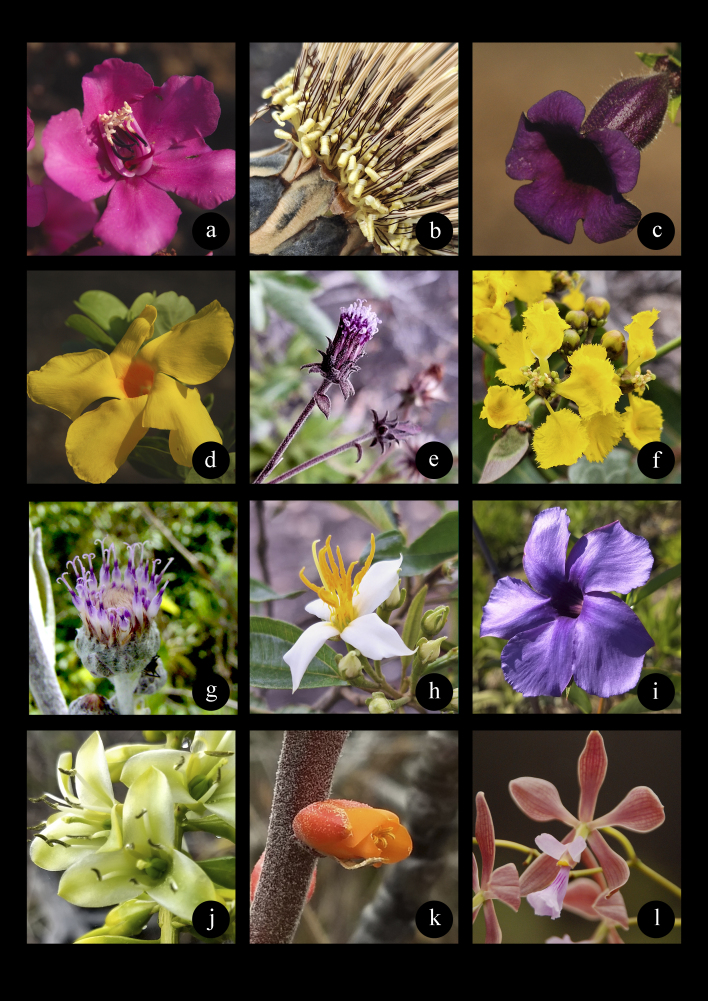
Species inhabiting vegetation islands on inselbergs in Espírito Santo State. **a** *Meriantherapulchra* Kuhlm.; **b** *Wunderlichiaazulensis* Maguire & G.M.Barroso; **c** *Sinningiaaghensis* Chautems; **d** *Mandevillafistulosa* M.F.Sales et al.; **e** *Cololobus* sp.nov.; **f** *Stigmaphylloncrenatum* C.E.Anderson; **g** *Cololobusargenteus* M.Monge & Semir; **h** *Huberiaespiritosantensis* Baumgratz; **i** *Mandevillagrazielae* M.F.Sales et al.; **j** *Dyckiahorrida* (L.B.Sm.) Forzza; **k** *Dyckiabracteata* (Wittm.) Mez; l *Encycliaspiritusanctensis* L.C.Menezes. All photos by Fabiula Arantes.

Our preliminary studies of the occurrences, based on online data for Pancas and Águia Branca, found there is a major sampling gap within the official limits of MONAPC, since all the collections are from a few points (Fig. 6). Many collections have the locality “*Monumento Natural dos Pontões Capixabas*”; however, the geographic coordinates are outside the official limits. This occurs because there is no clear physical delimitation of the natural monument limits, such as signs, which is probably because there is no management plan for the area. Additionally, most of the collections were made near the paved roads in the region and many of the collections are from the Três Pontões de Águia Branca Region due to a major collection effort during the work of Pinto-Junior et al. (2021). A few collections with coordinates within the Municipalities of Baixo Guandu and Nova Venécia were erroneously recorded for Pancas and Águia Branca. Localities with numerous collections, such as "*Pedra da Colina*" and "*Três Pontões de Águia Branca*", were not included in the protected area; they are a few kilometres outside the border of MONAPC. When MONAPC was being created, all these records would have been within the original planned area of 110,000 ha (Bortoleto 2015). However, in the official decree, only 17,000 ha were included in the protected area (15%).

## Usage licence

### Usage licence

Creative Commons Public Domain Waiver (CC-Zero)

## Data resources

### Data package title

Checklists

### Resource link

https://zenodo.org/doi/10.5281/zenodo.7831886

### Number of data sets

1

### Data set 1.

#### Data set name

Checklist of Rupicolous Vascular Plant Species in MONAPC

#### Data format

csv

#### Download URL

https://zenodo.org/doi/10.5281/zenodo.7831886

#### Description

Checklist of Vascular Plant Species in MONAPC (Monumento Natural dos Pontões Capixabas), Espírito Santo State, Brazil. It contains 108 species occurring on lowland inselbergs and highlights species included in official lists of endangered flora. Taxonomy, Life Form, Substrate, Vegetation Type, Occurrence Brazil and Domain information were downloaded from Flora e Funga do Brasil (2023) which are according to Instituto Brasileiro de Geografia e Estatística (2004).

**Data set 1.** 

Column label	Column description
Lineage	Descent of the taxon.
Family	Name of the family in which the taxon is classified.
Genus	Name of the genus in which the taxon is classified.
Epithet	Taxon specific epithet.
Author	Author of the monography for the taxon.
MMA 2022	Threat Status of the species according to Brazilian Red List: VU = Vulnerable, EN = Endangered, CR = Critically Endangered.
ES 2022	Threat status of the species according to Espírito Santo State Red List: VU = Vulnerable, EN = Endangered, CR = Critically Endangered.
Voucher	Indicates vouchers (collector and number).
Herbarium	Acronym of the herbarium according to Thiers (2023, continuously updated).
Code	Herbarium code of the voucher.
Life Form	Life form(s) that the taxon can exhibit: Herb, Shrub, Subshrub, Tree, Climbing, Sucullent, Subtree, Dracenoid.
Substrate	Place where the species occur: Rupicolous, Terrestrial, Epiphyte, Hemiepiphyte, Aquatic.
Vegetation Type	Vegetation type(s) where taxon is present: a = Área Antrópica [Anthropic Area], b = Cerrado (lato sensu), c = Floresta Estacional Decidual [Seasonally Deciduous Forest], d = Floresta Estacional Semidecidual [Seasonally Semideciduous Forest], e = Floresta Ombrófila Mista [Mixed Ombrophyllous Forest], f = Floresta Ombrófila (= Floresta Pluvial) [Ombrophyllous Forest (Tropical Rain Forest)], g = Vegetação Sobre Afloramentos Rochosos [Rock Outcrop Vegetation], h = Campo rupestre [Highland Rocky Field], i = Campo de Altitude [High Altitude Grassland], j = Restinga, k = Caatinga (stricto sensu), l = Carrasco, m = Campo Limpo [Grassland], n = Campo de Várzea [Flooded Field], o = Savana Amazônica [Amazonian Savannah], p = Campinarana, q = Floresta Ciliar ou Galeria [Riverine Forest or Gallery Forest], r = Floresta de Igapó [Inundated Forest (Várzea)], s = Floresta de Terra Firme [Terra Firme Forest], t = Vegetação Aquática [Aquatic Vegetation], u = Manguezal [Mangrove], v = Floresta de várzea [Inundated Forest (Várzea)], x = Floresta Estacional Perenifólia [Seasonal Evergreen Forest].
Occurrence Brazil	Brazilian States where taxon occurs: AC = Acre, AL = Alagoas, AM = Amazonas, AP = Amapá, BA = Bahia, CE = Ceará, DF = Distrito Federal, GO = Goiás, ES = Espírito Santo, MG = Minas Gerais, MA = Maranhão, MS = Mato Grosso do Sul, MT = Mato Grosso, PA = Pará, PB = Paraíba, PE = Pernambuco, PI = Piauí, PR = Paraná, RJ = Rio de Janeiro, RN = Rio Grande do Norte, RO = Rondônia, RR = Roraima, RS = Rio Grande do Sul, SC = Santa Catarina, SE = Sergipe, SP = São Paulo, TO = Tocantins.
Domain	Vegetation Domain where the taxon occurs: Ce = Cerrado, Ma = Mata Atlântica, Am = Amazônia, Ca = Caatinga, Pm = Pampa, Pa = Pantanal.
Complete Scientific Name	The full scientific name with author.
Scientific Name	Scientific name without author.
Origin	Indicate taxa that have originated in Brazil, with or without human envolvement (intentional or unintentional).
Endemism	Indicate taxa that only occur on inselbergs from southeast Brazil.

## Additional information

The flora of inselbergs has been neglected because these rocky outcrops are difficult to access and are commonly within anthropogenised matrices. The high levels of beta diversity (de Paula et al. 2021, Pinto-Junior et al. 2021), endemism and genetically differentiated populations (e.g. Barbará et al. (2007), Palma-Silva et al. (2011), Hmeljevski et al. (2015), Hmeljevski et al. (2017), Nazareno et al. (2020)) on rocky outcrops, in south-eastern Brazil, reinforce the fact that there is an insufficient number of inselbergs inside protected areas. MONAPC, in the heart of Sugarloaf Land, is a protected area in Brazil that contains a considerable number of lowland inselbergs. Therefore, we hope that this work contributes to the MONAPC management plan (Ministério do Meio Ambiente 2000), which will be challenging due to competing interests to farm locally and preserve the unique biota.

## Supplementary Material

8E9E9302-D000-5B9B-A497-ABCD09242972 10.3897/BDJ.12.e105688.suppl1 10490671 Supplementary material 1 Checklist of Rupicolous Vascular Plant Species in MONAPC Data type Checklist Brief description Checklist of Vascular Plant Species in MONAPC (Monumento Natural dos Pontões Capixabas), Espírito Santo State, Brazil. It contains 108 species occurring on lowland inselbergs and highlights species included in official lists of endangered flora. Taxonomy, Life Form, Substrate, Vegetation Type, Occurrence Brazil and Domain according to Flora e Funga do Brasil (2023). Endemic species from the granite inselbergs in southeast Brazil were determined using distribution data mainly taken from Flora e Funga do Brasil(2023) and/or by consulting papers about species description and their distribution. File: oo_954304.csv https://binary.pensoft.net/file/954304 Arantes, F.M.

## Figures and Tables

**Figure 5.**
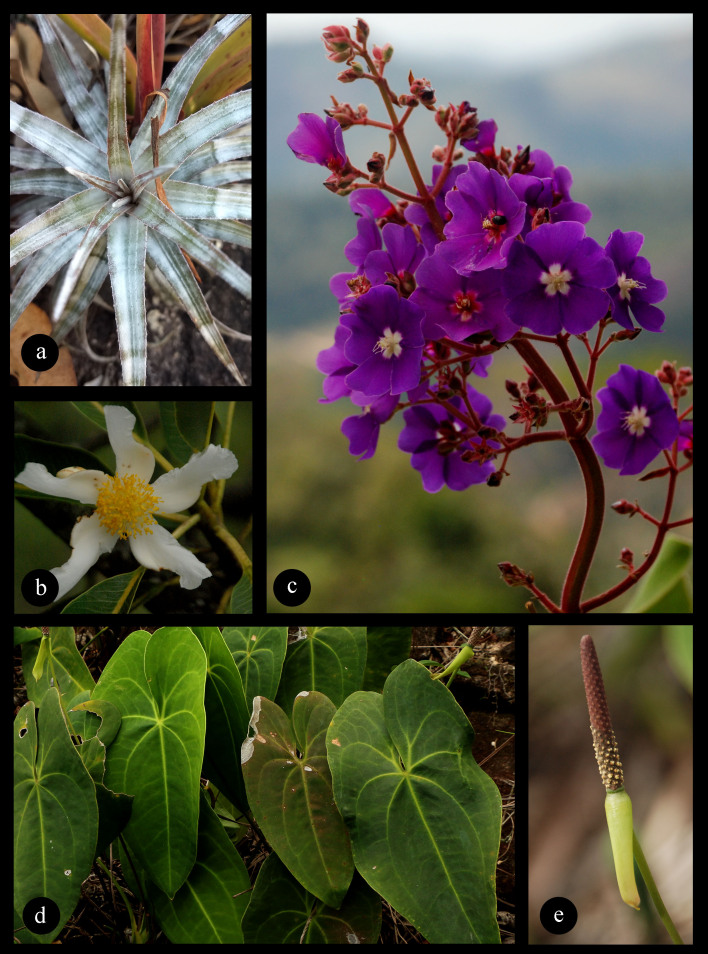
Species endemic to vegetation on inselbergs in Espírito Santo State. **a** *Orthophytumzanonii* Leme; **b** *Kielmeyerarupestris* Duarte; **c** *Pleroma* sp. nov.; **d, e** *Anthuriummarcusianum* Theoófilo, L.Kollmann & Sakur. All photos by Fabiula Arantes.

**Table 1.** Floristic studies conducted on inselbergs of Espírito Santo State, south-eastern Brazil. Rich. = Richness, F/G = Families/Genera, ES = microregions of Espírito Santo according to Instituto Jones dos Santos Neves (2021), Elev. = Elevation (m).

Study	Location	Rich.	F/G	ES	Elev.	Climate	Area
This study	MONAPC	108	36/75	Northeast	< 1000	Aw	17,000 ha
Pena and Alves-Araújo (2017)	APA Pedra do Elefante	302	74/219	Northeast	50–500	25ºC 800mm	2,562.31 ha
Esgario et al. (2009)	Alto Misterioso	170	44/109	Central	850–1143	No information	No information
Couto et al. (2017)	Pedra dos Pontões	211	51/130	South	700–1400	CwB 21ºC 1375mm	350 ha
Covre et al. (2021)	Pedra da Andorinha	121	40/96	South	150–500	CwA 1450mm	360 ha

**Table 2.** Inselbergs visited in MONAPC, Espírito Santo State, southeast Brazil.

#	Inselberg	Visited	Latitude	Longitude	Description
1	Pedra do Mirante	5	-19.055833	-40.741944	In front of Mrs. Dunalva and Mr. Tarcísio property, Pancas River's valley
2	Paredão Noroeste	2	-19.125617	-40.840733	Northeast Wall, São Luiz River's valley, near Mr. João Breda's property
3	Paredão das Ruschianas	2	-19.164444	-40.818611	Paranazinho River's valley
4	Sítio da Daiane	2	-19.061359	-40.745237	Near Mrs. Daiane's property
5	Sítio Fernando Oliosi	1	-19.189089	-40.841040	Near Mr. Fernando Oliosi's property, Paranazinho River's valley
6	Paredão Águia Branca	2	-19.061389	-40.745278	Unknown owner
7	Pedra da Mula	1	-19.175556	-40.792500	Near Pedra do Vidal
8	Sítio do Tiago	1	-19.022778	-40.684611	Near Mr. Tiago's property, Águia Branca
9	Sítio do Max Figueiras	1	-19.181528	-40.829694	Near Mr. Max Figueira's property, Paranazinho River's valley
10	Pedra do Vidal Krause	1	-19.190379	-40.782.088	Unknown owner

**Table 3.** Comparison of the number of species, genera and families amongst the different areas with floristic inventories of vegetation islands on inselbergs in Espirito Santo State.

Rank	This study (2021–23)	Covre et al. (2021)	Couto et al. (2017)	Pena and Alves-Araújo (2017)
Species	108	121	211	302
Families	36	40	130	219
Genera	74	96	51	74

**Table 4.** List of vascular plants in the "Monumento Natural dos Pontões Capixabas" classified in one of the threat categories, according to IUCN criteria (VU = Vulnerable, EN = Endangered, CR = Critically Endangered, NE = Not Evaluated), in the Brazilian Red List (Ministério do Meio Ambiente 2022) and in the Espírito Santo State Red List (Espírito Santo 2022).

Species	MMA2022	ES2022
*Alcantareasimplicisticha* Leme & A.P.Fontana	NE	VU
*Anemiapatens* Mickel & Labiak	NE	EN
*Anemiaretroflexa* Brade	NE	VU
*Axonopusgraniticola* P.L. Viana	NE	VU
*Bradeabrasiliensis* Standl.	EN	NE
*Cnidoscolushamosus* Pohl	CR	NE
*Cololobusargenteus* M.Monge & Semir	NE	EN
*Davillahirsuticarpa* Fraga & Aymard	NE	VU
*Dyckiabracteata* (Wittm.) Mez	NE	EN
*Dyckiacaudata* (L.B.Sm.)Forzza	NE	VU
*Dyckiahorrida* (L.B.Sm.) Forzza	EN	VU
*Encycliaspiritusanctensis* L.C.Menezes	NE	CR
*Epidendrumrobustum* Cogn.	VU	VU
*Huberiaespiritosantensis* Baumgratz	VU	NE
*Kielmeyerarupestris* Duarte	CR	CR
*Meriantheraburlemarxii* Wurdack	EN	EN
*Meriantherapulchra* Kuhlm.	VU	VU
*Orthophytumzanonii* Leme	CR	CR
*Pabstiellamuricatifolia* Fraga & L.Kollmann	NE	EN
*Peperomiaincana* (Haw.) Hook.	NE	EN
*Pitcairniabarbatostigma* Leme & A.P.Fontana	NE	VU
*Pitcairniadecidua* L.B.Sm.	EN	NE
*Pleromacucullatum* F.S.Mey., Fraga & R.Goldenb.	NE	CR
*Pleromafontanae* F.S.Mey., L.Kollmann & R.Goldenb.	NE	CR
*Pseudobombaxpetropolitanum* A.Robyns	EN	NE
*Pseudolaeliadutrae* Ruschi	VU	NE
*Sinningiaaghensis* Chautems	NE	VU
*Stachytarphetagesnerioides* Cham.	NE	EN
*Stigmaphylloncrenatum* C.E.Anderson	EN	EN
*Stigmatodonapparicianus* (E. Pereira & Reitz) Leme, G.K.Br. & Barfuss	NE	EN
*Stylosanthesguianensis* (Aubl.) Sw.	VU	NE
*Syagrusruschiana* (Bondar) Glassman	NE	VU
*Wunderlichiaazulensis* Maguire & G.M.Barroso	VU	NE

**Table 5.** Species described, based on type collections from the municipalities of Pancas and Águia Branca, for all vegetation types.

Family	Scientific name	Voucher	Collection Year	Municipality	Publication
Apocynaceae	*Mandevillagrazielae* M.F.Sales, Kin.-Gouv. & A.O.Simões	G.J. Sheperd 5869	1977	Águia Branca	Sales et al. (2006)
Araceae	*Anthuriummarcusianum* Théofilo, L.Kollmann & Sakur.	L. Kollmann 10937	2008	Águia Branca	Valadares et al. (2019)
Asteraceae	*Cololobusargenteus* M.Monge & Semir	A.P. Fontana 2330	2006	Águia Branca	Monge et al. 2018
Asteraceae	*Senecioespiritosantensis* A.M.Teles	H.Q. Boudet Fernandes 3457	2007	Águia Branca	Teles (2018)
Asteraceae	*Seneciohortensiae* A.M.Teles	A.P. Fontana 2344	2006	Pancas	Teles and Freitas (2013)
Begoniaceae	*Begoniaaguiabrancensis* L.Kollmann	V. Demuner 2286	2006	Águia Branca	Kollmann (2008)
Begoniaceae	*Begoniawasshauseniana* L.Kollmann & A.Peixoto	V. Demuner 3550	2007	Pancas	Kollmann and Peixoto (2012)
Bignoniaceae	*Adenocalymmaapetiolatum* L.H.Fonseca & Zuntini	H.Q. Boudet Fernandes 3508	2007	Águia Branca	Fonseca et al. (2016)
Bignoniaceae	*Adenocalymmalineare* L.H.Fonseca & Zuntini	L.F.S. Magnago 1158	2006	Nova Venécia	Fonseca and Lohmann (2019)
Bromeliaceae	*Alcantarealongibracteata* Leme & Fraga	E. Leme 7346	2008	Águia Branca	Versieux (2009)
Bromeliaceae	*Alcantareasimplicisticha* Leme & A.P.Fontana	E. Leme 7355	2008	Águia Branca	Versieux (2009)
Bromeliaceae	*Orthophytumpseudovagans* Leme & L.Kollmann	V. Demuner 2270	2006	Águia Branca	Leme and Kollmann (2007)
Bromeliaceae	*Orthophytumzanonii* Leme	A.P. Fontana 2324	2006	Pancas	Leme (2004)
Bromeliaceae	*Pitcairniabarbatostigma* Leme & A.P.Fontana	A.P. Fontana 2339	2006	Águia Branca	Leme et al. (2010)
Convolvulaceae	*Ipomoeascopulina* J.R.I.Wood & Scotland	D.P. Saraiva 47	2010	Águia Branca	Wood et al. (2017)
Dilleniaceae	*Davillahirsuticarpa* Fraga & Aymard	L.F.S. Magnago 1149	2006	Pancas	Fraga et al. (2017)
Dioscoreaceae	*Dioscoreamedusae* F.Fraga, R.Couto & J.M.A.Braga	F.R.M. Fraga 163	2017	Pancas	Fraga et al. (2019)
Flacourtiaceae	*Caseariasouzae* R.Marquete & Mansano	M.C.Souza 610	2008	Águia Branca	Marquete and Freitas Mansano (2010)
Leguminosae	*Senegaliagrazielae* M.J.F. Barros & M.P.Morim	V. Demuner 4783	2007	Águia Branca	Barros and Morim (2014)
Melastomataceae	*Meriantheraparvifolia* R.Goldenb., Fraga & A.P.Fontana	L.F.S. Magnago 1120	2006	Águia Branca	Goldenberg et al. (2012)
Melastomataceae	*Pleromamarinana* P.J.F.Guim. & Fraga	C.N. Fraga 962	2003	Águia Branca	Fraga and Guimarães (2014)
Melastomataceae	*Pleromapenduliflora* Fraga & P.J.F. Guim.	C.N. Fraga 965	2003	Pancas	Fraga and Guimarães (2014)
Myrtaceae	*Campomanesiasepalifolia* Luber & M.Ibrahim	J. Luber 230	2016	Águia Branca	Luber et al. (2017)
Myrtaceae	*Myrciacacuminis* L.Kollmann & Sobral	L.F.S. Magnago 1341	2006	Águia Branca	Sobral et al. (2014)
Orchidaceae	*Pabstiellamuricatifolia* Fraga & L.Kollmann	V. Demuner 2246	2006	Águia Branca	Fraga and Kollmann (2010)
Piperaceae	*Peperomiaaggregata* E.F.Guim. & Carv.-Silva.	M. Saavedra 684	2008	Águia Branca	Carvalho-Silva et al. (2019)
Rubiaceae	*Ixoraemygdioi* Di Maio & Peixoto	E.A. Bruno 191	1942	Águia Branca	Maio and Peixoto (2012)
Solanaceae	*Solanumfilirhachis* Giacomin & Stehmann	V. Demuner 4817	2007	Águia Branca	Knapp et al. (2015)
Violaceae	*Anchieteaballardii* Paula-Souza	D.P. Saraiva 48	2010	Águia Branca	Paula-Souza and Pirani (2016)

**Table 6.** List of endemics species under threat from inselbergs of southeast Brazil.

Family	Species	Reference
Apocynaceae	*Mandevillafistulosa* M.F.Sales et al.	de Sales et al. (2006)
Apocynaceae	*Mandevillagrazielae* M.F.Sales et al.	de Sales et al. (2006)
Apocynaceae	*Mandevillaobovata* J.F.Morales, A.P.Fontana & Fraga	Morales et al. (2022)
Araceae	*Anthuriummarcusianum* Theoófilo et al.	Valadares et al. (2019a)
Araceae	*Anthuriummicrophyllum* (Raf.) G.Don	Flora e Funga do Brasil (2023)
Araceae	*Anthuriummucuri* E.G.Gonç. & L.F.A.Paula	Flora e Funga do Brasil (2023)
Araceae	*Philodendronedmundoi* G.M.Barroso	Flora e Funga do Brasil (2023)
Arecaceae	*Syagrusruschiana* (Bondar) Glassman	Flora e Funga do Brasil (2023)
Asteraceae	*Cololobusargenteus* M.Monge & Semir	Monge et al. (2018)
Asteraceae	*Cololobuslongiangustatus* (G.M.Barroso) H.Rob.	Flora e Funga do Brasil (2023)
Asteraceae	*Wunderlichiaazulensis* Maguire & G.M.Barroso	Flora e Funga do Brasil (2023)
Bromeliaceae	*Alcantareanigripetala* Leme & L.Kollmann	Versieux (2009)
Bromeliaceae	*Alcantareasimplicisticha* Leme & A.P.Fontana	Versieux (2009)
Bromeliaceae	*Dyckiacaudata* (L.B.Sm.) Forzza	Leme et al. (2022) Gomes-Da-Silva et al. (2019)
Bromeliaceae	*Dyckiahorrida* (L.B.Sm.) Forzza	Leme et al. (2022) Gomes-Da-Silva et al. (2019)
Bromeliaceae	*Orthophytumfoliosum* L.B.Sm.	Flora e Funga do Brasil (2023)
Bromeliaceae	*Orthophytumzanonii* Leme	Leme (2004)
Bromeliaceae	*Pitcairniabarbatostigma* Leme & A.P.Fontana	Rocha et al. (2019)
Bromeliaceae	*Stigmatodonapparicianus* (E.Pereira & Reitz) Leme et al.	Leme et al. (2022)
Bromeliaceae	*Stigmatodonvellozicolus* (Leme & J.A.Siqueira) D.R.Couto & A.F.Costa	Leme et al. (2022)
Cactaceae	*Coleocephalocereusfluminensis* (Miq.) Backeb.	Flora e Funga do Brasil (2023)
Calophyllaceae	*Kielmeyerarupestris* Duarte	Flora e Funga do Brasil (2023)
Euphorbiaceae	*Cnidoscolusurentissimus* Fern.Casas	Fernández-Casas (2003)
Malpighiaceae	*Stigmaphylloncrenatum* C.E.Anderson	Flora e Funga do Brasil (2023)
Melastomataceae	*Huberiaespiritosantensis* Baumgratz	Flora e Funga do Brasil (2023)
Melastomataceae	*Meriantheraburlemarxii* Wurdack	Goldenberg et al. (2012)
Melastomataceae	*Meriantherapulchra* Kuhlm.	Goldenberg et al. (2012)
Melastomataceae	*Pleromacucullatum* F.S.Mey. et al.	Flora e Funga do Brasil (2023)
Melastomataceae	*Pleromafontanae* F.S.Mey. et al.	Flora e Funga do Brasil (2023)
Melastomataceae	*Pleromamarinanum* P.J.F. Guim. & Fraga	Fraga and Guimarães (2014)
Melastomataceae	*Pleromapenduliflorum* Fraga & P.J.F.Guim.	Fraga and Guimarães (2014)
Orchidaceae	*Encycliaspiritusanctensis* L.C.Menezes	Bastos et al. (2018)
Orchidaceae	*Pabstiellamuricatifolia* Fraga & L.Kollmann	Fraga and Kollmann (2010)
Orchidaceae	*Pseudolaeliadutrae* Ruschi	Menini Neto et al. (2013)
Poaceae	*Axonopusgraniticola* P.L. Viana	Viana and de Paula (2013)
Rubiaceae	*Bradeabrasiliensis* Standl.	Flora e Funga do Brasil (2023)
Turneraceae	*Oxossiarubrobracteata* (Arbo) L.Rocha	Rocha et al. (2019)
Velloziaceae	*Velloziacandida* J.C.Mikan	Mello-Silva (2004)
